# Editorial

**Published:** 1869-08

**Authors:** 


### Editorial.

New Mercy Hospital.—The corner stone of the new Mercy Hospital building was laid, with due ceremony, on the 25th of July. A very large number of citizens were present, and interested in the exercises. The public address, together with a description of the building, will be given in the September number of the Examiner.

College Lecture Fees.—We see it stated in some of the medical journals that the American Medical Association, at its recent annual meeting, adopted resolutions declaring the minimum fee for a regular college lecture term should be $120, and that such colleges as refused to comply with that rate should be denied representation in said Association. The statement, however, is an error. The Association did adopt a simple declaration of opinion, that the *minimum fee* for a regular college lecture term should be $120. It did *not* adopt any resolution in relation to denying representation to such colleges as did not accept the standard proposed. And, so far as we have learned, the colleges generally are issuing their circulars with the same rate of fees for the coming session as had been required during the past year. The action of the Association was in the form of a simple expression of opinion, and nothing more.

Medical Controversies.—Perhaps there is no one thing better calculated to impair the honor and usefulness of the medical profession, than the bitter personal controversies, or, more properly speaking, *quarrels*, that every now and then spring up among its members, and find ventilation either through the medical or secular press.

Some men appear to be so constituted that they cannot correct the errors, refute the arguments, or point out the misrepresentations of others, without directly attacking their personal characters. Such men are continually acting as though the most violent denunciation constituted the most conclusive argument, and the blackening of the character the most complete correction of all misrepresentations. They are practically incapable of discussing any subject, either on the platform or through the press, without involving themselves in *personalties* altogether foreign to the subject itself.

These thoughts have been suggested chiefly by an article in a recent number of the *Nashville Medical and Surgical Journal*, by T. S. Bell, M.D., of Louisville, concerning E. S. Gaillard, M.D., of the same city. From this article it would appear that Dr. Bell had at some previous time delivered and published a lecture on a subject relating to antiquarian researches. Dr. Gaillard, in his capacity of editor of the *Richmond and Louisville Medical Journal*, reviews this lecture, pointing out what he supposed to be defects in its style and logic.

Ostensibly for this Dr. Bell writes a dozen pages of the most violent personal abuse of Dr. Gaillard; and what is perhaps even less excusable, the editor of the *Nashville Medical Journal* permits the article to appear in his columns. We say less excusable, because the editor, as a third party, is supposed to be free from the personal feeling that actuates the writer, and consequently capable of seeing the injurious bearing of such papers, both on the writer and on the profession at large. If he had returned the manuscript to Dr. Bell, with the friendly advice to burn it up, or reduce it to a simple dignified correction of whatever errors or misstatements he supposed to have been made in the *Richmond and Louisville Journal*, how much of censure and scandal would have been saved to all parties.

Viewed in the light simply of a reply to the review of his lecture in the *Richmond and Louisville Medical Journal*, the article of Dr. Bell, in the *Nashville Journal*, is in the highest degree censurable. If the editor of a medical journal cannot exercise the right of noticing the publications of the day, and in such notices pointing out their defects or errors, or both, without calling out in return such a torrent of personal detraction as we find in the article of Dr. Bell, then is the position of a medical editor a very undesirable one indeed. But the truth seems to be, that the review in the *Richmond and Louisville Journal* only served Dr. Bell for a pretext, the real cause of his article being a bitter feud which had existed many months between Drs. Bell, Gaillard, and others connected with the two medical colleges in Louisville; a feud which had found vent in the newspapers of that city, and which the profession generally will regard as reflecting no credit upon any of the parties concerned.

We can excuse men for getting into a passion when assailed unexpectedly in conversation, but to dip a pen into gall and sit deliberately down to expose the predominence of our passions over our judgment, is to show the weak side of poor human nature unnecessarily.

---

"Sweet Quinine."—The preparation largely advertised under this name has been analyzed by the editor of the *American Journal of Pharmacy*, who states that it is not quinine at all, "but mainly the alkaloid *cinchonia* precipitated from the sulphate, dried and triturated with an impure glycyrrhizin prepared from liquorice root," in the proportion of about three parts of cinchonia to one of inpure glycyrrhizin. The *Journal* adds: "Cinchonia, however tasteless, is not quinine, nor does its commercial value approach that of quinine so nearly as it is made to do in the garb of 'sweet quinine.' When physicians want cinchonia they can get it by prescription, and it is not in accordance with our ideas of fair dealing to serve it up as a new substance."—*N. Y. Med. Gazette.*

---

Cholera and Yellow Fever in Cuba.—Sickness is increasing in the ranks of the Spanish and rebel forces, and the mortality is frightful. It is estimated that the deaths amount to 15 per cent. monthly, of the men in the field on both sides. The Spanish troops are afflicted with vomito, while the insurgents suffer from cholera and diarrhœa, caused especially by want and exposure. When the hot and rainy seasons are past the volunteers will go into active service in the field and reinforcements will arrive from Spain.—*Havana, July 17th.*

The Messrs. Johnson, of Philadelphia, announce an important work on "*The Jurisprudence of Medicine in its relations to the Law of Contracts, Torts, and Evidence*," by Professor John Ordronaux. Such a work has long been needed, and the author's reputation gives assurance that the hiatus will be thoroughly filled.—*N. Y. Med. Gazette.*

---

Impurity of Water.—In every 100,000 tons of the water supplied to London, the solid impurity averages from twenty-eight to forty-two tons; in Edinburgh, from eleven to fourteen tons; Bristol, twenty-eight tons; Manchester, six tons; Dublin, six tons; and Glasgow, only three tons.—*Ibid.*

---

The Contagion of Consumption.—M. Chauveau, Professor in the Lyons Veterinary School, continues perseveringly his researches on the contagiousness of Tuberculosis. He has of late selected the intestinal surface as the field for his investigations, and through it by introducing tuberculous matter into the circulatory current he has produced at will general tubercle. The *Union Medicale* reports that he lately purchased four handsome heifers, and he tuberculized three of them by causing them to swallow 30 grammes each of tuberculous matter taken from the body of an old phthisical cow. The rapidity of the result was extraordinary. At the end of twenty days the first heifer had lost flesh to a surprising extent, its pulse was quick and full, and coughed incessantly. At the end of fifty-two days it was killed, and it presented perfectly marked tuberculous lesions, situated especially about the mesentry and intestine. The mesenteric glands presented infiltrarion in so high a degree that many were of the size of the first. Their total mass weighed 1650 grammes. All the ganglia of the bronchi and mediastinum were enlarged, and the lung was full of crude tubercle. The other two heifers presented not less perfectly marked alterations, while the fourth, to which none of the tuberculous matter had been administered, remained intact and increased in flesh.

It is proved, therefore, that animals of the bovine species contract tuberculosis by digestive ingestion, just as they take carbuncle and cowpock, as sheep take the rot, as the horse takes glanders, and as man takes small-pox. The human digestive tube constitutes an easy channel for tuberculous contagion. If bovine phthisis be identical with tuberculosis in the human species, there is, in the use of the flesh of tuberculous animals, a danger to which the poor are more especially exposed.—*Med. Press and Circular.*

MORTALITY FOR THE MONTH OF JUNE, 1869:—

CAUSES OF DEATH.

| | |
|---|---|
| Accidents, burned, | 1 |
| " drowned, | 5 |
| " concussion of brain, | 1 |
| " in plaining mill, | 1 |
| " poisoned, | 1 |
| " injuries from knife, | 1 |
| " railroad, | 5 |
| " swallowing foreign substance, | 1 |
| " scalded, | 1 |
| Abscess-pelvic, | 1 |
| Apoplexy, | 7 |
| Asphyxia, | 2 |
| Births, premature, | 23 |
| " still, | 40 |
| Bowels, inflammation, | 4 |
| Brain, congestion of, | 3 |
| " disease of, | 2 |
| " inflammation, | 11 |
| " & lungs, " | 1 |
| " softening of, | 1 |
| Bronchitis, | 3 |
| " hepatitis & icterus, | 1 |
| " capillary, | 1 |
| Breast, cancer of, | 2 |
| Cerebritis, | 1 |
| Cholera infantum, | 14 |
| Convulsions, | 33 |
| Consumption, | 37 |
| Croup, | 1 |
| " membranous, | 1 |
| Cyanch, maligna, | 1 |
| Debility, | 2 |
| " & convulsions, | 1 |
| Delirium tremens, | 3 |
| Diarrhœa, | 7 |
| " chronic, | 4 |
| Diphtheria, | 2 |
| Dropsy, | 6 |
| " abdominal, | 1 |
| Dysentery, | 6 |
| " & convulsions, | 1 |
| Embrolism of pulmonary artery from phlebitis, | 1 |
| Entero-colitis, | 1 |
| Enteritis, | 1 |
| Erysipelas, | 1 |
| " of face, | 1 |
| Fever, congestive, | 1 |
| " puerperal, | 5 |
| " remittent, | 1 |
| " scarlet, | 50 |
| " " and hydrocephalus, | 1 |
| " typhoid, | 12 |
| " typhus, | 2 |
| Gastritis, | 4 |
| " and bowels inflammation, | 1 |
| Hæmoptysis, | 1 |
| Heart, disease of, | 4 |
| " valvular " | 1 |
| Hernia, incarcerated, | 1 |
| Hydrothorax, | 1 |
| Hydrocephalus, | 9 |
| " acute, | 2 |
| Inanition, | 13 |
| Intemperance, | 2 |
| Kidneys, Bright's disease of, | 1 |
| " and cystitis, | 1 |
| Larynx, stricture of, | 1 |
| Laryngitis, | 1 |
| Liver, congestion and inflammation with uremia, | 1 |
| " softening of, | 1 |
| Lungs, congestion of, | 2 |
| " emphysema of, | 1 |
| Measles, | 31 |
| " and diarrhœa, | 2 |
| Meningitis, cerebro-spinal, | 3 |
| " tubercular, | 3 |
| Nutrition, defective, | 1 |
| Old-age, | 5 |
| Paralysis, | 3 |
| Putella, fracture of, | 1 |
| Peritonitis, | 1 |
| " puerperal, | 2 |
| " traumatic, | 1 |
| Pharynx, cancer of, | 1 |
| Pneumonia, | 22 |
| " and measles, | 1 |
| " broncho, | 3 |
| " typhoid, | 2 |
| Rheumatism, inflammatory, | 1 |
| Scrofula, | 1 |
| Spine, fracture of, | 1 |
| Shock from injuries, | 1 |
| Suicide, | 2 |
| Small pox, | 2 |
| Tabes mesenterica, | 8 |
| Teething, | 7 |
| " and convulsions, | 5 |
| " and diarrhœa, | 3 |
| Tetanus, traumatic, | 1 |
| Tongue, cancer of, | 1 |
| Thrush, | 1 |
| Urine, suppression of, | 1 |
| Vitality, deficient, | 1 |
| Whooping-cough, | 8 |
| " and pneumonia, | 1 |
| Unknown, | 5 |
| Total, | 434 |

COMPARISON.

Deaths in June, 1869, 434 | Deaths in June, 1868, 305 | Increase, 129

Deaths in May, 1869, 372 | Increase, 62

AGES.

| | |
|---|---|
| Under 1 | 122 |
| 1 to 3 | 96 |
| 3 to 5 | 36 |
| 5 to 10 | 35 |
| 10 to 20 | 14 |
| 20 to 30 | 36 |
| 30 to 40 | 31 |
| 40 to 50 | 21 |
| 50 to 60 | 23 |
| 60 to 70 | 10 |
| 70 to 80 | 8 |
| 80 to 90 | 0 |
| 90 to 100 | 1 |
| Unknown | 1 |
| Total, | 434 |

| | | |
|---|---|---|
| Males, 241 | Females, 193 | Total, 434 |
| Single, 334 | Married 100 | Total, 434 |
| White, 429 | Colored, 5 | Total, 434 |

NATIVITY.

| | | | | | |
|---|---|---|---|---|---|
| Bohemia, | 4 | Germany, | 56 | Scotland, | 3 |
| Canada, | 6 | Holland, | 7 | Sweden, | 37 |
| Chicago, Native, | 61 | Ireland, | 38 | Switzerland, | 1 |
| Chicago, Foreign, | 132 | New Brunswick, | 1 | Wales, | 1 |
| U. S., other parts, | 56 | Norway, | 17 | Unknown, | 2 |
| Denmark, | 3 | Poland, | 1 | | |
| England, | 7 | Prince Edward Island | 1 | Total, | 434 |

MORTALITY BY WARDS FOR THE MONTH.

| Wards. | Mortality. | Pop. in 1868. | One death in |
|---|---|---|---|
| 1 | 8 | 9,094 | 1,136¾ |
| 2 | 18 | 13,074 | 726⅓ |
| 3 | 21 | 15,076 | 718 |
| 4 | 21 | 17,796 | 847½ |
| 5 | 29 | 16,033 | 552 5-6 |
| 6 | 18 | 13,083 | 726 5-6 |
| 7 | 40 | 25,492 | 637⅓ |
| 8 | 30 | 15,813 | 523⅔ |
| 9 | 25 | 19,297 | 772 |
| 10 | 7 | 12,925 | 1,846½ |
| 11 | 18 | 14,340 | 796⅔ |
| 12 | 36 | 17,485 | 485⅔ |
| 13 | 11 | 11,164 | 1,015 |
| 14 | 14 | 14,839 | 1,052¾ |
| 15 | 29 | 21,078 | 726 4-5 |
| 16 | 13 | 15,465 | 1,189⅔ |

| Mortality. | |
|---|---|
| Unknown Ward, | 1 |
| Accidents, | 17 |
| County Hospital, | 10 |
| Home for Friendless, | 3 |
| Immigrants, | 42 |
| Mercy Hospital, | 4 |
| Protestant Orphan Asylum, | 2 |
| St. Luke's Hospital, | 1 |
| St. Joseph Orphan Asylum, | 13 |
| Suicide, | 2 |
| Police Station, | 1 |
| Total, | 434 |

